# Software application profile: the Rapid Inquiry Facility 4.0: an open access tool for environmental public health tracking

**DOI:** 10.1093/ije/dyz094

**Published:** 2020-04-15

**Authors:** Frédéric B Piel, Brandon Parkes, Peter Hambly, Aina Roca-Barceló, Martin McCallion, Giovanni Leonardi, Heather Strosnider, Fuyuen Yip, Paul Elliott, Anna L Hansell

**Affiliations:** 1 UK Small Area Health Statistics Unit (SAHSU), Department of Epidemiology & Biostatistics, School of Public Health, Imperial College London, London, UK; 2 MRC-PHE Centre for Environment & Health, Department of Epidemiology & Biostatistics, School of Public Health, Imperial College London, London, UK; 3 Environmental Epidemiology Group, Centre for Radiation, Chemical and Environmental Hazards, Public Health England, Chilton, UK; 4 Environmental Public Health Tracking Program, National Center for Environmental Health, Centers for Disease Control and Prevention, Atlanta, US; 5 Centre for Environmental Health and Sustainability, University of Leicester, Leicester, UK

**Keywords:** Disease mapping, risk analysis, surveillance, cluster detection, epidemiology

## Abstract

The Rapid Inquiry Facility 4.0 (RIF) is a new user-friendly and open-access tool, developed by the UK Small Area Health Statistics Unit (SAHSU), to facilitate environment public health tracking (EPHT) or surveillance (EPHS). The RIF is designed to help public health professionals and academics to rapidly perform exploratory investigations of health and environmental data at the small-area level (e.g. postcode or detailed census areas) in order to identify unusual signals, such as disease clusters and potential environmental hazards, whether localized (e.g. industrial site) or widespread (e.g. air and noise pollution). The RIF allows the use of advanced disease mapping methods, including Bayesian small-area smoothing and complex risk analysis functionalities, while accounting for confounders. The RIF could be particularly useful to monitor spatio-temporal trends in mortality and morbidity associated with cardiovascular diseases, cancers, diabetes and chronic lung diseases, or to conduct local or national studies on air pollution, flooding, low-magnetic fields or nuclear power plants.

**Key Messages**

- There is a growing need for tools enabling rapid environment public health surveillance.
- The Rapid Inquiry Facility (RIF) 4.0 is an open-source software tool aiming to facilitate disease mapping and risk analysis epidemiological studies.
- The RIF 4.0 facilitates the analysis of health, demographic, socio-economic and environmental data with sophisticated space-time statistical methods.

## Introduction

There is growing pressure on national and international public health institutions to develop robust disease surveillance systems. Such systems help routinely monitor and evaluate the health of the populations for which they are responsible. They also generate the evidence required to inform cost-effective interventions and prevention policies. Such systems rely on the continued analysis and monitoring of the distribution and trends of disease incidence through the systematic collection, consolidation, analysis and dissemination of epidemiological data in public health practice.^1^ The need for robust surveillance systems is well-established for outbreaks of infectious diseases (also termed communicable diseases), which allow successful public health interventions. For example, the rapid measures implemented by public health authorities of the Democratic Republic of the Congo to successfully contain local Ebola outbreaks in 2017 and 2018, were largely praised internationally.^2^ Despite the fact that many countries, including the UK, have excellent and comprehensive surveillance systems for most infectious diseases, the Zika epidemic that affected South America in 2015–2016 revealed fault lines in the world’s collective preparedness, including the ability to track cases of congenital birth defects potentially linked to Zika infections.

Conducting surveillance for non-communicable diseases (NCDs) is also part of the remit of national and international public health institutions.^3^ NCDs include common conditions such as cardiovascular and respiratory diseases, cancers and diabetes, as well as rare ones including, for example, congenital anomalies. Developing systems for NCD surveillance has hitherto been largely neglected, although this area has received more attention in the last few years.^4^ The proportion of the global burden of diseases attributable to NCDs has been rapidly increasing. Based on the 2016 iteration of the Global Burden of Disease (GBD) study, NCDs have become the leading cause of mortality and morbidity worldwide, increasing from 44% of the disability-adjusted life years (DALYs)—a combined measure of mortality and morbidity—in 1990 to 61% in 2016.^5^ This shift is well documented in high-income countries^6^ and evidence from low- and middle-income countries is now rapidly growing.^7^

Alongside systems implemented strictly for disease surveillance, identifying early risk factors potentially affecting the health of local populations, such as environmental hazards (e.g. air pollution, water and soil contamination, radiation, climate change), also falls within the remit of most public health institutions. For example, the 2017 Annual Report of the Chief Medical Office focused on the threat to health posed by pollution to people living in England.^8^ The conclusions of *The Lancet* Commission for Pollution and Health^9^ highlighted that the impact of environmental factors on health are considerable and that diseases caused by pollution accounted for 16% of all deaths worldwide in 2015.

**Figure 1.**
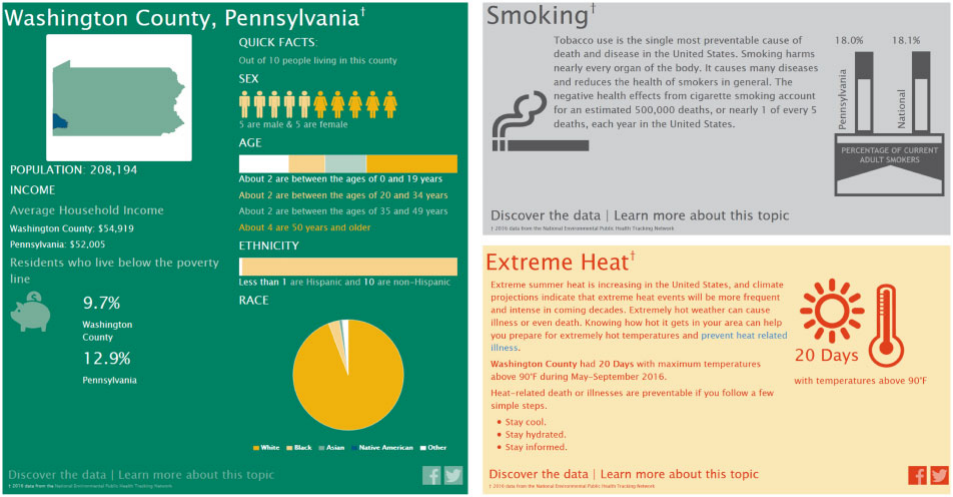
Snapshot of some of the environmental health issues presented in the *Info by Location* tool of the CDC’s Environmental Public Health Tracking Program for the county of Washington, PA. The infographics also include data on asthma, heart attacks, air quality (ground-level ozone and particulate matter), access to parks and proximity to highways (not shown).

Environmental Public Health Tracking (EPHT) has been defined as the ongoing collection, integration, analysis and interpretation of data about environmental hazards, exposure to environmental hazards and human health effects potentially related to exposure to environmental hazards. It includes dissemination of information learned from these data analyses and interpretation.^10^ One of the best-established EPHT systems worldwide is the National Environmental Public Health Tracking Program developed by the US Centers for Disease Control and Prevention (CDC). Set up following a report from the Pew Environmental Health Commission in 2000,^11^ CDC’s EPHT Program provides timely, accurate and systematic environmental data to both public health decision makers and members of the public in order to protect the nation from health issues arising from or directly related to environmental factors, and to reduce the environmental health burden. This is achieved by effectively linking environmental health data and translating it into meaningful information to protect the health of the public. Successful case studies of the EPHT programme have been described in detail in Eatman and Strosnider (2017).^12^ In 2013, the CDC launched the *Info by Location* online portal allowing users to visualize a range of data on demographics, health conditions and environmental risk factors in their local area (Figure 1). The Data Explorer (https://ephtracking.cdc.gov/DataExplorer/) is another interactive tool of CDC’s EPHT Network that offers visualization of multiple measures on asthma, cancers, heart disease and other diseases, as well as air quality, climate change, heat stress illness.

In England, Public Health England (PHE) is currently working on an EPHT programme aimed at exploring and developing a national methodology for addressing environmental hazards. Once operational, PHE’s Environmental Public Health Surveillance System (EPHSS) (https://www.gov.uk/government/publications/environmental-public-health-surveillance-system) will deliver integrated, local and national surveillance of environmental hazards, exposure assessment and related health effects of exposure to those hazards. EPHSS builds on the experience and expertise developed through the design and operation of related systems currently used by PHE and other UK government agencies to facilitate the collection and collation of environmental hazard and health outcome data. Examples of ‘hazard tracking’ already conducted by EPHSS include the monitoring of 500 private water supplies for arsenic and other chemicals through biomonitoring,^13^ the assessment of public health impacts of fluoridation of public water supplies,^14^ and the characterization of the burden of disease from carbon monoxide (CO) poisoning.^15^

To support the development of these surveillance systems, appropriate tools are essential. CDC and PHE have long recognized this need, as reflected by ongoing collaborations with the UK Small Area Health Statistics Unit (SAHSU) (http://www.sahsu.org/) and other research units to support the development of software such as the Rapid Inquiry Facility (RIF) (https://www.sahsu.org/content/rapid-inquiry-facility).

## Implementation

The RIF is a dedicated analytical software system aimed at considerably reducing the time required by researchers or public health professionals to investigate potential public health risks. It provides a powerful tool to link health, demographic and environmental data; to evaluate spatial relationships between different data sources; to explore and visualize the data through disease mapping; and to calculate health risks in relation to sources of environmental pollution. It can dramatically speed up data analysis and public health inquiries such as those conducted by surveillance systems (e.g. investigation of potential disease clusters).

The RIF was first developed in the late 1990s by SAHSU within the Department of Epidemiology and Biostatistics at Imperial College London, UK. SAHSU has a national four-fold remit to: (i) develop methodologies, particularly for small areas; (ii) improve the detection of health risks from pollution; (iii) conduct targeted research and monitoring; and (iv) advise government on unusual clusters of disease. SAHSU holds hundreds of millions of records from routinely collected national health databases, alongside environmental and socio-demographic data. The RIF was originally designed as a tool for SAHSU staff to routinely analyse collected health data in relation to environmental exposures in the UK.^16^

Between 2000 and 2003, the RIF was transformed for use by seven European partners as part of the European Health and Environment Information System (EUROHEIS) project (http://www.euroheis.orf).^17^ This project demonstrated the usefulness of the RIF beyond SAHSU in answering questions concerning environmental health risks, utilizing the system within the context of improving public health, preventing human illness and diseases, and obviating sources of danger to health in the UK, Denmark, Finland, Ireland, Italy, Spain, Sweden and The Netherlands.^18^

Since 2005, CDC and SAHSU have collaborated on adapting and enhancing the RIF software for use in CDC’s National EPHT Network (https://www.cdc.gov/nceh/tracking/partners/sahsu.htm). The goal of this collaboration is to increase the functionality and versatility of the RIF for use in evaluating temporal and spatial relationships between disease and environmental hazards in the National EPHT Network. Through the use of a more user-friendly interface, the RIF could be made available to additional countries and widely disseminated via the Internet, including across low- and middle-income countries.

### Previous versions of the RIF

Previous RIF versions were originally designed as an embedded extension of the geographical information system (GIS) ArcMap 8.0 and ArcGIS 9.x (ESRI, Redlands, CA, USA) connected to an external database (such as Microsoft Access or Oracle) of geocoded health and population data. ArcGIS is one of the most widespread GISs worldwide and many public health and academic institutions have licences. The most widely used RIF version (3.x) has been employed by more than 45 institutions and public health practitioners across 25 countries both to automatically generate disease maps and to assess disease risk in proximity to known sources of pollution. Nevertheless, the costs involved with licensing ArcGIS limited access to the RIF, particularly in low- and middle-income countries.

By tightly linking a GIS and a database, the RIF removed the need to explicitly gather data study by study, saving on both time and data storage. The value of the RIF (3.x) has been previously illustrated with case studies of risk of leukaemia in areas surrounding oil refineries in the State of Utah, USA and of the geographical variation of risk of oesophageal cancer in relation to zinc cadmium sulphide exposure in Norwich, UK.^17^ More recent examples reflecting the international range of users of the RIF include a geo-mapping study of time trends in childhood caries risk in Sweden^19^; an analysis of the relationship between statin utilization and socio-economic deprivation in Hungary^20^; and disease mapping of cancers in Ontario, Canada.^21^

### The RIF 4.0

**Figure 2.**
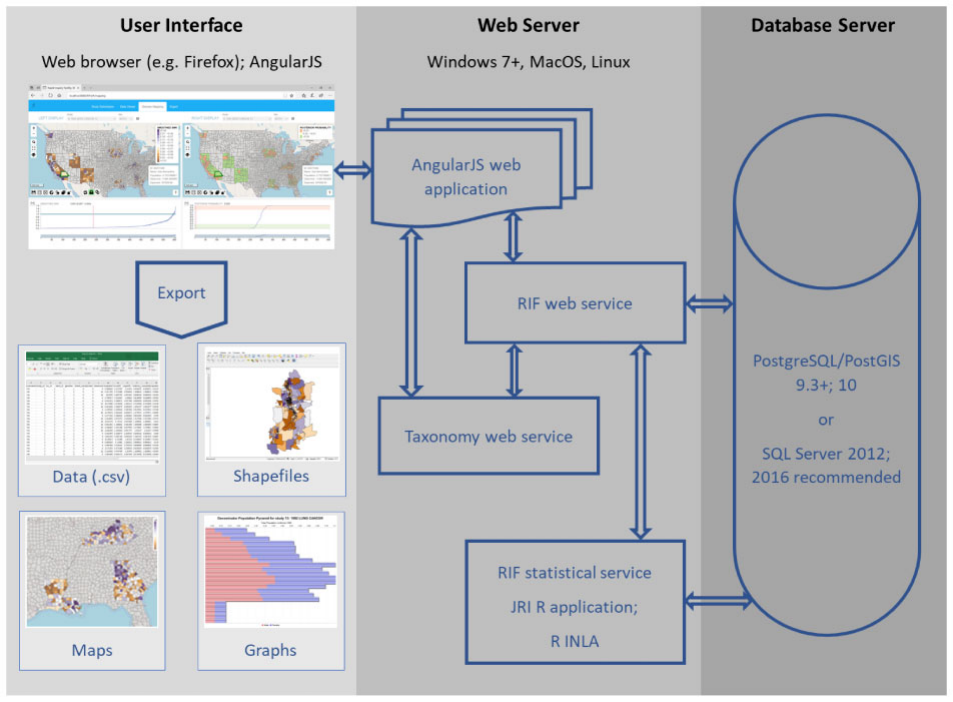
Overview of the architecture of the RIF 4.0. The user uses the RIF through a web browser. The web server interacts with the database server via SQL queries which are customized to handle the data type, as well as syntactical and functional differences between Postgres and SQL Server. AngularJS is a JavaScript-based open-source front-end web application framework that permits the development of well-structured web applications; the INLA approach approximates Bayesian inference for latent Gaussian models by using integrated nested Laplace approximations; JRI: Java *R* interface allows the statistical service to use R; PostGIS is an open-source software program that adds support for geographic objects to the Postgres; Postgres is an open-source object-relational database management system (ORDBMS) with an emphasis on extensibility and standards compliance; *R* is a programming language and free software environment for statistical computing and graphics supported by the R Foundation for Statistical Computing; RIF Web Service is the principal provider of services to AngularJS; RIF Statistical Service uses *R* and *R INLA* to calculate the RIF results; shapefiles are a popular geospatial vector data format for geographic information system (GIS) software; SQL Server is a relational database management system developed by Microsoft; the Taxonomy Web Service provides taxonomies such as ICD 9 and ICD 10 lookup to AngularJS and the RIF Web Service.

The RIF 4.0 is a complete redesign of the RIF 3.2, building on the strengths of previous versions and removing some of their limitations. The RIF 4.0 has been redeveloped as a open-source project, independent of ArcGIS and licensing fees, that can be used on-line or off-line in a range of web-browsers, including Chrome, Firefox and Internet Explorer. The open-source licensing should contribute to (i) making it available to a broader range of users, particularly in low- and middle-income countries; (ii) allowing software developers from all around the word to contribute improvements; and (iii) enabling regular independent audits. The RIF 4.0 uses a three-layer architecture composed of the user interface (or client), the web server and the database server (Figure 2). The RIF user interface uses Leaflet (https://leafletjs.com/) for mapping. Leaflet is designed for simplicity, performance and usability. The resolution of maps depends on the scale, ensuring fast draw rates, and this gives the RIF the ability to visualize health outcomes on screen even when mapping small geographical areas across a large region or country, such as the counties at a continental US scale. All data can be stored locally or remotely on a spatially enabled database that is directly linked to a Java middleware. The role of the middleware is to check, validate and secure all communications between the user interface, a JavaScript/HTML5 platform, and the database. The RIF database can be implemented in either PostgreSQL/PostGIS or Microsoft SQL Server to suit the requirements of most users. Postgres offers a powerful, open-source object-relational database system, while MS SQL Server provides a reliable and readily approved environment for many public health institutions. The RIF 4.0 integrates advanced methods in statistics, exposure assessment and data visualization. It is based on open-source software integrated with statistical packages running in *R*^22^ with the ability to read in local sources of environment and health data for data analysis.

Detailed documentation and all the code underlying the development of the RIF 4.0 are freely accessible on GitHub (https://smallareahealthstatisticsunit.github.io/rapidInquiryFacility/) to allow users or developers to find further information, track progress on ongoing developments and potentially contribute to future improvements.

The RIF has two core functionalities: disease mapping and risk analysis. For each of these, it is essential to carefully assess the most appropriate scale of investigation (i.e. the study area and the reference area). This will depend on local circumstances (i.e. population density) and on the frequency of the health outcome of interest (i.e. common or very rare). These decisions are often based on a compromise between data availability, the smallest resolution possible and the stability of estimates. When investigating health risks in small areas, the number of observed and expected cases in local populations may be small, which leads to unstable estimates and misleading maps or risk maps. This is particularly important when studying rare diseases. Surveillance of exposure to pollutants affecting health and reliably detecting spatio-temporal signals in NCD data (e.g. ‘clusters’, peaks or unusual trends) rely both on high-quality data and on the use of advanced statistical methods. Being able to detect areas of potentially high risk of specific NCDs requires methods that offer both specificity (i.e. few false positive findings) and sensitivity (i.e. high ability to detect true positives). Apparent local clusters of disease may, after investigation (see, for example, recent cluster guidelines published by PHE),^23^ indicate areas with higher-quality data registration or areas where there are many duplicate registrations. Results from an epidemiological study might only apply to a certain portion of the population based on, for example, the size of the study area, the nature of the environmental risks, or the local socio-economic context. Differentiating real signals from false positives is therefore an important methodological challenge. Surveillance of chronic diseases has so far mostly focused on specific conditions (e.g. hepatic angiosarcoma, mesothelioma, leukaemia), rather than on a generic approach to detecting excesses or anomalies in the data.

## Core functionality 1: Disease mapping

The disease mapping functionality can be used to visualize rates (e.g. mortality/morbidity) and risks across the study area. Disease mapping can provide an invaluable tool to explore spatial patterns of health outcomes; identify potential issues regarding data quality by geographical area; and identify areas that need additional resources or remediation.

Age standardization adjusts for differences in disease risk that might result from different age structures in small areas (e.g. older population vs younger population). This will particularly affect diseases that are strongly associated with age. For example, without adjustment, inner city wards that have a much lower proportion of older residents might appear to have a lower risk of a cardiovascular disease or cancer when compared with rural wards that have higher proportions of older residents. In such a case, some or possibly all of the difference would be an artefact due to the differences in age structure of the population.

By default, the RIF calculates standardized mortality (or incidence/morbidity) ratios (SMRs). Unlike previous versions of the RIF, the RIF 4.0 only supports indirect standardization, rather than both direct and indirect standardizations. This is because, at the small geographical level, the number of cases studied is usually so few that directly standardized rates are unstable and the imprecision of this measure makes comparisons very difficult. In such situations, it is appropriate to use SMRs provided that the stratum-specific death rate for each exposure class is proportional to the standard population rates, and bearing in mind that the rates in each exposure group may not be directly comparable with each other.^24^

**Figure 3.**
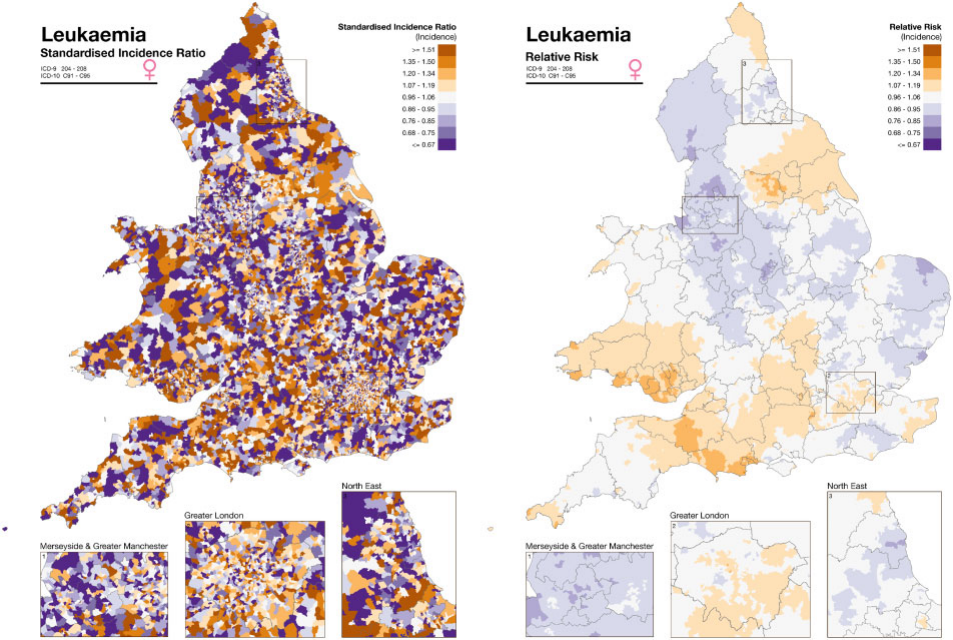
Illustration of the disease mapping approach and empirical Bayesian smoothing of the RIF 4.0 as originally developed for the SAHSU Environment and Health Atlas for England and Wales (http://www.envhealthatlas.co.uk/homepage/).^30^ Disease mapping of leukaemia in females in England and Wales with standardized incidence ratio (left) and smoothed relative risk (right). The data on the left are very noisy and no underlying pattern can be discerned. Smoothing shows that the incidence of leukaemia is not random but there is a slowly varying underlying geographic pattern that can be readily visualized.

The RIF 4.0 also performs empirical Bayesian smoothing. Smoothing is a statistical method used to adjust for chance fluctuations in disease risk that can occur when risks are calculated using small numbers of cases or small populations. Smoothing of the raw relative risks accounts for sampling variability in the observed data and may reveal patterns from otherwise noisy maps (Figure 3). Three Bayesian smoothing options are currently implemented in the RIF 4.0: (i) Poisson- lognormal (HET); (ii) intrinsic conditional auto-regressive (ICAR); and (iii) Besag, York and Mollié (BYM).^25–27^ The HET model involves smoothing across the whole study area (‘global’ smoothing), whereas the ICAR model uses ‘local’ smoothing by borrowing information from neighbouring areas. In the BYM model, an additional unstructured spatial random effect is included to account for independent region-specific noise in order to combine ‘local’ and ‘global’ smoothing. While such Bayesian models originally relied on computationally expensive Markov chain Monte Carlo techniques, the recent development of the integrated nested Laplace approximation (INLA) approach, to which SAHSU contributes, and its integration in an *R* package (R-INLA) greatly facilitate their use.^28^^,^^29^

The smoothed risk estimates are calculated with 95% confidence intervals, but another measure of uncertainty is provided by the posterior probability which indicates the probability that the relative risk is >1. Maps showing the posterior probability allow the user to interpret the strength of the statistical evidence of excess risk in the study areas.^30^ Further help to interpret relative risks and uncertainty is available in Section 4 of the Sense about Science publication ‘Making Sense of Statistics’ (http://www.senseaboutscience.org/data/files/resources/1/MSofStatistics.pdf) and in Professor Spiegelhalter’s article ‘2845 ways to spin the risk’ (http://understandinguncertainty.org/node/233).

## Core functionality 2: Risk analysis

The risk analysis functionality of the RIF 4.0 can be used to explore whether a pollution source or some particular environmental exposure is having an impact on health in the population of a local area. To carry out a risk analysis study, the user first needs to carefully consider the following. (i) The geographical position of the putative risk factor. In GIS terms, this can typically be a point (e.g. a mobile phone aerial), a line (e.g. a road or a powerline) or an area (e.g. a plume or a contaminated area). (ii) The distance within which the exposure of interest is expected to have an impact. It might be appropriate to consider different levels of exposure (e.g. high vs low) and to use multiple sub-study areas. (iii) The duration of the exposure as this will determine which years of health data are required for the study. The availability of a few years of data will likely be sufficient to explore a short-term effect, whereas several decades of data could be required to study long-term effects.

**Figure 4.**
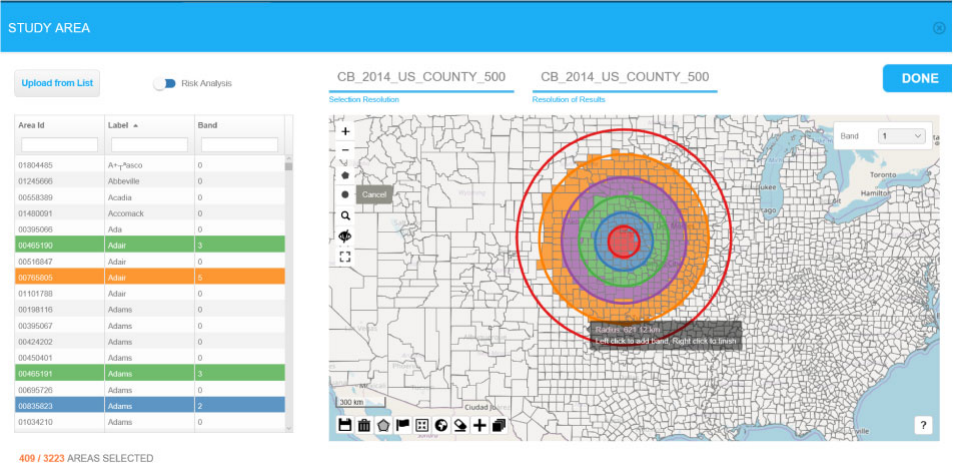
Illustration of the risk analysis functionalities of the RIF 4.0. The maps show selected small area (counties) falling within successive concentric buffers (100, 200, 300, 450 and 600 km) drawn around one possible local source of pollution in the USA. Areas are selected based on the location of population-weighted centroids.

The RIF allows users to map the location of one or multiple sources of exposure (e.g. all the incinerators or nuclear power plants in a given country), or to import a file containing their locations. Various tools, including concentric buffers (Figure 4) or outputs from dispersion models, can then be used to define the level of exposure. This can be done either based on evidence from the literature or on field measurements. Areas can be selected based on the geographic centroid or the population-weighted centroid of each area. The RIF performs tests on the relative risk to assess for homogeneity and linear trend with exposure and provides graphs of the risks as a function of exposure per band.

## Usage

### Installation, data loading and outputs

Detailed instructions about the RIF 4.0 build and installation process are available on GitHub (https://smallareahealthstatisticsunit.github.io/rapidInquiryFacility/). The installation is streamlined to minimize the effort required. Participating in the RIF development requires familiarity with Java web applications. Larger non-desktop or laptop installations require database and system administration experience to handle the data volumes and secure network setup. The basic installation of the RIF 4.0 will initially only include hierarchical geographies for the UK and the USA. Geographies for other countries are likely to be added at later stages or can be added by users themselves.

To conduct a study, the RIF requires the following types of data. (i) Numerator data. This may be in individual record form or aggregated to a suitable administrative geography. Individual records are assumed to have been de-identified into a suitable pseudonymous form, although this will depend on the characteristics of the system on which the RIF is installed (e.g. private vs public network). (ii) Denominator data (e.g. census data). (iii) Covariate data (e.g. socio-economic status in quintiles). (iv) An administrative geography which is created by the Tile-Maker tool.

The RIF data loading occurs in two consecutive phases: pre-processing and RIF load processing. The pre-processing phase aims to ensure that the data is formatted in a format compatible with the RIF. The end products are CSV files suitable for use in a RIF load script. Examples of scripts for both Postgres and SQL Server using the US SEER Cancer Registry data are available in the RIF documentation. The RIF load processing loads the new data on the RIF database, resulting in a fully configured RIF with the data loaded. Typically, users need to be granted access to the dataset to be able to use the data. Data without access is not visible to users in the RIF.

The RIF 4.0 outputs include both detailed data tables and maps presenting (i) the population and health data of each geographical area included in the study area (including Area Id, Band Id, Observer, Population, investigation Id); and (ii) basic statistics (adjusted and unadjusted expected, counts, relative risk with confidence intervals) and the results of the Bayesian smoothing (posterior probability, smoothed SMR with confidence intervals). These outputs are primarily generated as simple text files (e.g. CSV) and can be further processed with any statistical or GIS software.

## Conducting a study

**Figure 5.**
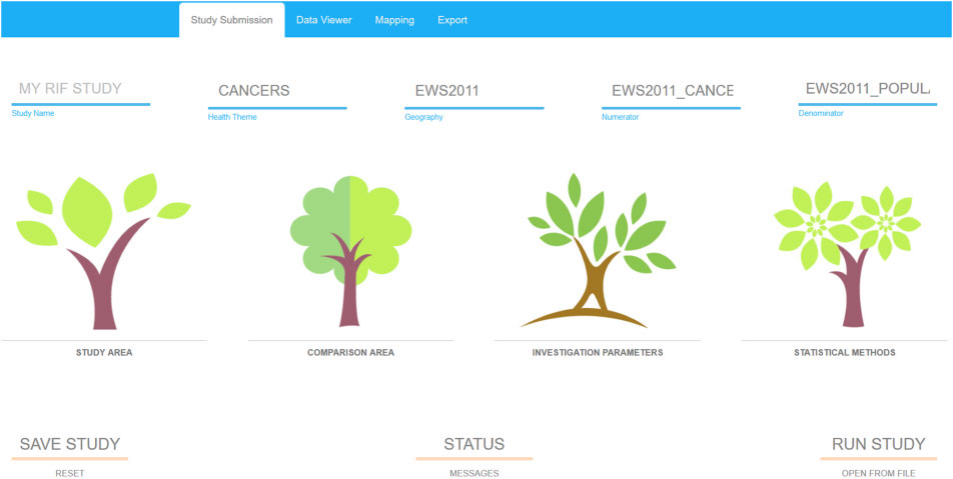
Screenshot illustrating the 4 consecutive steps involved in conducting a RIF 4.0 study: defining the study area, defining the comparison area, setting the investigation parameters and choosing the statistical methods to be used.

**Figure 6.**
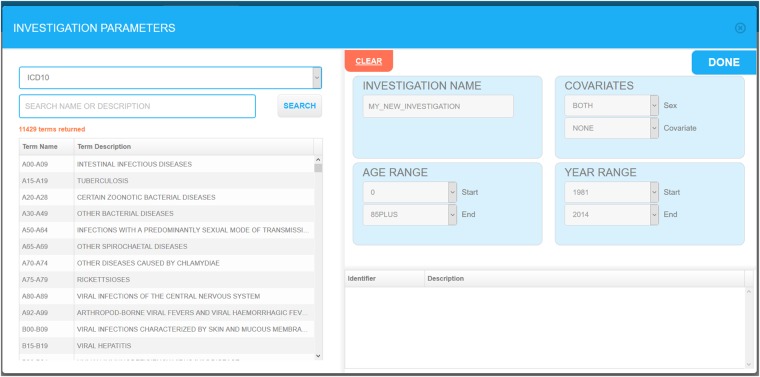
RIF study investigation parameters selection screen.

A RIF study is based on four successive user-friendly steps: (i) defining the study area; (ii) defining the comparison area; (iii) setting investigation parameters such as the ICD codes, age, sex and confounder relevant to the study; and (iv) choosing the statistical smoothing method to be used (Figure 5). Most choices can be done through simple drop-down menus (Figure 6). Studies can be saved at any stage and parameters can be modified to run a new study. Outputs include a range of simple text files and standard shapefiles which can be further analysed using specialized statistical or GIS software. Further guidance is available in an online demo on the SAHSU website (https://www.sahsu.org/content/rapid-inquiry-facility/disease-mapping-demo).

## Limitations

Disease mapping and risk analysis studies, including those conducted at small-area level, are complex and require a systematic approach to assess and avoid biases in data, as well as in their analysis and interpretation. As described in our accompanying paper,^31^ careful attention should be paid to data quality and a series of careful and informed methodological choices are required at each stage of a study. Although, the RIF 4.0 has been designed as a user-friendly tool, the accuracy and relevance of the outputs will depend on the rigour with which these choices have been made by the user.

## Conclusion

The RIF 4.0 is a unique piece of software to support environment public health surveillance systems by allowing users to rapidly monitor spatio-temporal trends in the mortality and morbidity associated with common diseases such as cardiovascular diseases, cancers, diabetes and chronic lung diseases, as well as rare diseases, or to conduct local or national studies, for example, on air pollution, flooding, low-magnetic fields or nuclear power plants. The RIF 4.0 is scalable and can rapidly process vast amounts of data. With the appropriate IT support and the detailed documentation available, the RIF can be installed in an institution’s network infrastructure to provide exploratory disease mapping and risk analysis functionalities to a wide range of health professionals and researchers. The RIF provides user management functionality based on widely used, scalable databases, allowing the RIF to be integrated within existing information governance frameworks. Furthermore, the data persistency and export options mean it is straightforward to use the results of exploratory investigations as a starting point for complex epidemiological studies. Although some of the functionalities of the RIF 4.0 can be performed with other specialist software, these alternatives tend to require an expensive licence (e.g. ArcGIS), or specific coding skills (e.g. *R* packages) or lack multi-user persistence and scalability (e.g. SpatialEpi *R* package) (https://cran.r-project.org/web/packages/SpatialEpi/index.html).

As illustrated by key collaborations with PHE and CDC and by the range of users of previous versions of the RIF, there is a demand for tools and methods facilitating the conduct of surveillance activities through disease mapping and risk analysis. With attention on the burden of NCDs and environmental risk factors on health growing globally and developing data collecting efforts in low- and middle-income countries, it is likely that the need for open access and user-friendly software such as the RIF will continue to increase. To maximize the potential of such tools, NCD surveillance needs further investments to help develop strategies for disease prevention and for the detection and treatment of those already affected. Emerging space-time surveillance methods, such as BaySTDetect,^32^ along with machine learning and other computing intensive data science methods, offer potential to carry out such analyses using a systematic approach. These methods could ultimately be added to the RIF functionalities to provide early warning systems of any untoward trends in the national health datasets.

## Funding

The UK Small Area Health Statistics Unit (SAHSU) is part of the MRC-PHE Centre for Environment and Health, which is supported by the Medical Research Council (MR/L01341X/1) and Public Health England (PHE). We acknowledge support from the NIHR Health Protection Research Unit in Health Impact of Environmental Hazards (HPRU-2012–10141). Part of this study was supported by a Wellcome Trust Seed Award in Science (204535/Z/16/Z) awarded to F.B.P. Funding from the US Center for Disease Control (CDC) as part of the US Environment and Health Public Tracking Program (http://www.cdc.gov/nceh/tracking) was also received.

## Notes

The RIF 4.0 has been developed as open source software under the GNU Lesser General Public License. Detailed instructions about its source code, build and installation process are freely available on GitHub at (https://smallareahealthstatisticsunit.github.io/rapidInquiryFacility/).
